# Pre-Hospital Factors Influencing Time of Arrival at Emergency Departments for Patients with Acute ST-Elevation Myocardial Infarction

**DOI:** 10.21315/mjms2019.26.1.8

**Published:** 2019-02-28

**Authors:** See Choo Lim, Andey Rahman, Najib Majdi Yaacob

**Affiliations:** 1 Department of Emergency Medicine, School of Medical Sciences, Universiti Sains Malaysia, 16150 Kubang Kerian, Kelantan; 2 Unit of Biostatistics and Research Methodology, School of Medical Sciences, Universiti Sains Malaysia, 16150 Kubang Kerian, Kelantan

**Keywords:** emergency medicine, pre-hospital delay, ST-elevation myocardial infarction, symptom-to-door time, factors

## Abstract

**Background:**

Pre-hospital delay is currently a major factor limiting early reperfusion among ST-elevation myocardial infarction (STEMI) patients worldwide. This study aims to determine pre-hospital factors affecting symptom-to-door time among STEMI patients in Malaysia.

**Methods:**

This cross-sectional study included 222 STEMI patients admitted to two tertiary hospitals in Malaysia. By determining symptom-to-door time, the study population was categorised into two definitive treatment seeking groups: early (≤ 3 h) and delayed (> 3 h). Data was collected focusing on socio-demographical data, risk factors and comorbidities, clinical presentation, situational factors and action taken by patients.

**Results:**

The mean age of our patients was 58.0 (SD = 11.9) years old, and the population consisted of 186 (83.8%) males and 36 (16.2%) females. Our study found that the median symptom-to-door time was 130.5 (IQR 240) min, with 64% of subjects arriving early and 36% arriving late. Pre-hospital delays were found to be significant among females (adj OR = 2.42; 95% CI: 1.02, 5.76; *P* = 0.046), patients with recurrence of similar clinical presentations (adj OR = 2.74; 95% CI: 1.37, 5.46; *P* = 0.004), patients experiencing atypical symptoms (adj OR = 2.64; 95% CI: 1.11, 6.31; *P* = 0.029) and patients who chose to have their first medical contact (FMC) for their symptoms with a general practitioner (adj OR = 2.80; 95% CI: 1.20, 6.56; *P* = 0.018). However, patients with hyperlipidaemia (adj OR = 0.46; 95% CI: 0.23, 0.93; *P* = 0.030), self-perceived cardiac symptoms (adj OR = 0.36; 95% CI: 0.17, 0.73; *P* = 0.005) and symptoms that began in public places (adj OR = 0.21; 95% CI: 0.06, 0.69; *P* = 0.010) tended to seek treatment earlier.

**Conclusion:**

The symptom-to-door time among the Malaysian population is shorter in comparison to other developing countries. Nevertheless, identified, modifiable pre-hospital factors can be addressed to further shorten symptom-to-door time among STEMI patients.

## Introduction

Acute myocardial infarction is a leading cause of mortality worldwide (1). In Malaysia, the 2006–2013 National Cardiovascular Disease Database-Acute Coronary Syndrome (NCVD-ACS) registry showed a significant increase in the number of patients admitted for acute coronary syndrome (ACS)—from 3,392 in 2006 up to 6,127 in 2013. Among the spectrum of ACS, ST-segment elevation myocardial infarction (STEMI) has remained the most common, with 50.8% of total ACS patients from 2011–2013 (2). Unfortunately, only 75% of all STEMI patients were eligible to receive thrombolytic therapy, and only 9.4% were eligible to receive primary percutaneous coronary intervention (PCI). The main reasons for this ineligibility for revascularisation were late arrival at emergency departments and doctors having missed the diagnosis of STEMI (2), both of which lead to increased mortality and morbidity.

Acute management of STEMI patients emphasises early reperfusion with either thrombolytic therapy or primary percutaneous coronary intervention (PCI); this plays an utmost important role in reducing infarct size, preserving left ventricular function, and improving STEMI patients’ short- and long-term outcomes (3). However, delayed reperfusion treatment is a global issue, and every 30 min delay increases the risk of one-year mortality by 7.5% (4). Research has shown that the greatest benefit from reperfusion therapy occurs in patients treated within one hour of symptom onset, with a sharp decline in efficacy when treatment is delayed for 3 h after symptom onset (5). According to the 2014 Malaysia Clinical Practise Guideline (CPG) on the management of STEMI, the best reperfusion therapy choice depends on the time frame of patients arriving at the emergency department. For early arrival patients (within 3 h of symptom onset), both thrombolytic therapy and primary PCI demonstrate almost equal efficacy; for late arrival patients (3–12 h), primary PCI is preferable for a better outcome. For very late arrival patients (more than 12 h), both primary PCI and thrombolytic therapy are not routinely recommended in patients who are asymptomatic and hemodynamically stable, unless the patient has persistent ischemic chest pain or is experiencing hemodynamical and electrical instability; then, primary PCI is preferable. Regarding modes of reperfusion, primary PCI has established its superiority over thrombolytic therapy (6). However, in Malaysia, only 9.4% of patients from 2011 to 2013 received PCI, most likely due to its limited availability in public hospitals. Most patients received thrombolytic therapy instead (2). Therefore, the timeline for reperfusion is particularly crucial for STEMI patients in hospitals with limited access to primary PCI, where thrombolytic therapy is the chosen mode of reperfusion.

To shorten time to reperfusion, the Ministry of Health Malaysia has carried out strategies to minimise pre- and in-hospital delays for STEMI patients. There is expansion of pre-hospital emergency services, rapid transfer for suspected STEMI cases to the nearest hospital with thrombolytic therapy, as well as targeted door-to-needle (DTN) and door-to-balloon (DTB) times that must be achieved according to the latest STEMI guidelines (2014). Although DTN and DTB times have significantly shortened recently (2), pre-hospital delay or symptom-to-door times have been overlooked, and there is limited data available concerning them in Malaysia. Recent studies in different countries have highlighted the various factors associated with pre-hospital delays, asserting that they are major contributors to delayed treatment seeking among STEMI patients (7–11). However, there is limited information regarding pre-hospital delay factors in Malaysia. Therefore, the aim of this study is to determine the symptom-to-door times of STEMI patients among the Malaysian population and to analyse variables associated with early (≤ 3 h) and late (> 3 h) hospital arrivals by looking at patients’ socio-demographical data, risk factors and comorbidities, symptom characteristics, situational factors and action taken by patients in seeking treatment. Identification of these factors is important for raising awareness and improving public education.

## Materials and Methods

### Patients and Setting

This cross-sectional study was conducted between 15 December 2016, and 23 January 2018, in two tertiary care hospitals in two states: Hospital Universiti Sains Malaysia (HUSM), Kubang Kerian, Kelantan, Malaysia; and Hospital Sultanah Nur Zahirah (HSNZKT), Kuala Terengganu, Terengganu, Malaysia. HUSM received around 120 STEMI patients per year, and HSNZKT received approximately 250 STEMI patients per year. The study population consisted of 222 patients from the emergency departments of both hospitals, who were diagnosed with STEMI and fulfilled the following study criteria.

### Study Criteria

Patients were included in this study if they were aged > 18 years old, had developed acute STEMI outside of the hospital, with known times of symptom onset, and had been diagnosed with STEMI by treating physicians under at least two of the following conditions: 1) typical or atypical chest pain and associated symptoms, 2) elevated serum cardiac biomarkers and 3) ST elevation on electrocardiography (ECG) in two contiguous leads. The cut-off point for new, or presumed to be new, ST segment elevation [in the absence of left ventricular hypertrophy (LVH) and left bundle branch block (LBBB)] was the presence of ≥ 0.1 mV ST segment elevation in all leads, except for leads V2–V3. In leads V2–V3, a cut-off point of ≥ 0.25 mV (in males < 40 years), ≥ 0.2 mV (in males ≥ 40 years) and ≥ 0.15 mV in females were used.

### Sampling Method and Sample Size Calculation

This study applied a purposive sampling method. Using Najib’s formula software for sample size calculation for comparing two proportions (12), the largest sample size calculated was 222, based on available literature reviews.

### Data Collection

Once diagnosis was established, the patients were first treated and stabilised in the Emergency and Trauma Department (ETD) and were subsequently admitted to the Cardiac Care Unit (CCU) followed by the Cardiac Rehabilitation Ward (CRW). Patients were interviewed once stabilised in the ETD, CCU or CRW, within seven days after first arriving at the emergency department. Patients were interviewed using proforma, focusing on:

- Socio-demographical data: age, gender, education level.
- Risk factors and comorbidities: hypertension, diabetes mellitus, family history of coronary artery disease (CAD), hyperlipidaemia, smoking.
- Symptom characteristics:
  - Typical symptoms: heavy retrosternal chest pain radiating to the jaw and shoulder, profuse sweating, nausea and vomiting, shortness of breath.
  - Atypical symptoms: dyspepsia, lethargy, loss of consciousness, feeling of weakness, absence of any typical symptoms.
  - Chest pain intensity using a numerical rating scale [0–10], categorised into no pain [0], mild pain [1–3], moderate pain [4–6] and severe pain [7–10].
  - Self-perceived cardiac symptoms, which indicate the patient’s self-awareness that the symptoms he or she experienced were related to heart diseases.
  - Recurrence of symptoms (i.e., patient had a history of equivalent symptoms).
- Situational factors: place of symptom onset, time of symptom onset and arrival time at ETD.
- Action taken: first medical contact (FMC), means of transportation to the hospital.

From the data collected, symptom-to-door time—defined as the time interval between symptom onset and time of arrival at the ETD offering thrombolytic therapy or a facility with access to primary PCI—was calculated in minutes and categorised into two groups: early arrival patients (symptom-to-door time ≤ 3 h) and delayed arrival patients (symptom-to-door time > 3 h) groups. For patients who were intubated, terminally ill or passed away during admission, interviews were conducted with the patient’s family members, who were able to provide the necessary information. Each interview session lasted approximately 30 min.

### Statistical Analysis

Statistical analysis was performed using IBM SPSS for Windows, version 24.0 (SPSS Inc. Chicago, IL, USA). Symptom-to-door time in minutes was calculated by subtracting the patient’s ETD arrival time from the time of symptom onset. Categorical data were expressed as numbers and percentages, while continuous data were expressed as mean and standard deviation (SD) or median and interquartile range (IQR). As the results were skewed, symptom-to-door times were described using medians and interquartile ranges (IQR). Each variable was tested for association with early arrival patients (≤ 3 h or 180 min) and delayed arrival patients (> 3 h or 180 min), using the simple logistic regression model. Variables with *P* < 0.25 were subsequently subjected to multiple logistic regression analysis, and 13 variables were considered for inclusion in this model. All these variables were then analysed using backward and forward selection procedures to select the final, optimal model. Adjusted odds ratios (adj OR) and 95% confidence intervals (CI) were reported, and *P* < 0.05 was considered statistically significant.

## Results

A total of 222 patients were recruited for this study, with a mean age was 58.0 (SD = 11.9) years old and a range of 28 to 85 years old. Among the patients, 186 (83.8%) were male and only 36 (16.2%) were female. The overall median symptom-to-door time was 130.5 (IQR 240) min. Of all patients, 142 (64%) arrived early and 80 (36%) delayed their arrivals.

The baseline characteristics of patients who arrived early and those who delayed are presented in Table 1. Out of all male patients, only 30.6% arrived late, compared to 63.9% of all female patients. In the simple logistic regression analysis, which is presented in Table 2, we found that being female, having hypertension, presenting atypical symptoms, and choosing to have FMC about the symptoms with a general practitioner were factors associated with a higher risk of delay. On the contrary, patients who smoked, experienced severe chest pain, had their symptoms begin in public places and self-perceived their symptoms to be cardiac related had shorter symptom-to-door times.

Results of the multiple logistic regression are presented in Table 3. Female patients were found to be associated with 2.42 times higher odds of delayed arrival than males (*P* = 0.046). Patients with recurrent symptoms also tended to delay 2.74 times more, compared to patients who were first experiencing chest pain (*P* = 0.004). In addition, atypical symptoms were associated with 2.64 times higher odds for pre-hospital delays than typical symptoms (*P* = 0.029). Regarding FMC, patients who first sought treatment at a General Practitioner Clinic exhibited 2.80 times higher odds of prolonging symptom-to-door time compared to patients who first sought treatment directly at an emergency department or a government health clinic (*P* = 0.018). Among all the risk factors and comorbidities, patients with hyperlipidaemia had 54% lower odds of delaying hospital arrival (*P* = 0.030). Patients’ self-perceptions of their cardiac symptoms also significantly affected symptom-to-door time—lowering the odds of delay by 64% (*P* = 0.005). Concerning the places of symptom onset, patients who first experienced symptoms in public places had 79% lower odds of delaying than patients whose symptoms occurred at home or in their workplaces (*P* = 0.010).

## Discussion

In our study, median symptom-to-door time was 130.5 min, which is comparable to developed countries, such as Australia, with a pre-hospital delay time of 132 min (11), the United States (US) with 162 min (9), Korea with 150 min (13) and Beijing, China, with 110 min to 140 min (10, 14). Our median time was approximately 50% shorter compared to some developing countries, such as India (ranging from 250 min to 290 min) (7, 15). This finding is consistent with the higher proportion of early arrival (64%) among our STEMI patients. This shorter symptom-to-door time may be due to the widespread healthcare facilities available in our country in both rural and urban areas. District hospitals, with 24-h emergency services, are equipped with thrombolytic therapy, and, during office hours, health clinics are easily accessible with on-call medical staff readily available. Furthermore, pre-hospital care (PHC) units have been established in Malaysia since 2013, with improved Medical Emergency Response Systems (MERS 999), especially in coordinating emergency calls and despatching ambulance services efficiently via the Medical Emergency Coordinating Centre (MECC). However, this result may be biased by the non-probability sampling.

### Socio-Demographic Factors

In our study, the female group was found to have a higher tendency for delaying emergency department arrival compared to male patients. This is similar to data found in the 2011–2013 NVCD-ACS registry, in which females had longer pain-to-needle time, with non-significant differences of door-to-needle time, compared to males (2). Previous studies also found that being a female is an independent predictor of delayed emergency department arrival among STEMI patients (11, 16–18). This is probably due to the prevalence of atypical symptom presentations, with non-chest pain, among the female group (17). Lawesson et al. (17) found there was insufficient knowledge among female patients regarding the clinical presentation of acute myocardial infarction (AMI), so they could not accurately interpret their symptoms as being cardiac related (17). In addition, females were found to traditionally consider STEMI as a ‘male disease’ (19), hence further mitigating their suspicion of having myocardial infarction.

### Risk Factors and Comorbidities

Among the risk factors investigated, only hyperlipidaemia was found to be statistically significant in multivariate analysis, with lower odds for pre-hospital delays. In previous studies, conflicting results were reported for hypertension, diabetes mellitus, family history of heart disease, smoking and hyperlipidaemia as predictors to delayed emergency department arrival (9, 20, 21). However, our study has shown that these (except hyperlipidaemia) were insignificantly associated with prolonged pre-hospital delays, while patients with underlying hyperlipidaemia were found to be associated with shorter symptom-to-door times. This finding can be compared to a study conducted in Srinagar, India, in which the absence of dyslipidaemia was significantly associated with prolonged pre-hospital delays (7). It is well known, in our society, that hyperlipidaemia is highly related to cardiovascular diseases, including myocardial infarction. This perception may explain patients’ earlier decisions to seek treatment, as they probably associated their seemingly cardiac related symptoms with their coexisting hyperlipidaemia.

### First Medical Contact

Although 68.5% of our studied patients first sought treatment directly at an emergency department without ‘doctor shopping’, 16.2% of STEMI patients’ FMCs were general practitioners (GP), and this decision was significantly associated with delays in arriving at an emergency department for definitive treatment (*P* < 0.001). Previous studies also reported similar findings, in which initially contacting GPs further delayed symptom-to-door time (9, 19, 22, 23). Most GP clinics in Malaysia are not well equipped with ECG machines to detect STEMI, and this may lead to a patient’s symptoms being misinterpreted as benign conditions and being subsequently misdiagnosed. In such cases, patients will likely be sent home with oral medications to wait for symptoms to be alleviated.

### Clinical Characteristics

From our study, 18% of patients presented atypical symptoms, and they had 2.64 times higher odds of delayed emergency department arrival. In agreement with our findings, atypical symptoms, such as fatigue and non-chest pain, were reported as significant predictors for pre-hospital delays (9, 11). Atypical symptoms were frequently perceived as benign and non-cardiac in origin (24), hence the lack of urgency to seek treatment earlier in a hospital. Our society may be aware of typical angina symptoms, such as crushing retrosternal chest pain with classical Levine’s sign, but many still lack awareness regarding atypical presentations that may occur, especially among females and among diabetic patients with neuropathy.

Garofalo et al. (25) reported that pre-hospital delay was mainly determined by the time spent making the decision to call an ambulance, with a median of 2 h elapsed (25). Decision making time significantly depends on patients’ cognitive factors and their intuitive understanding about myocardial infarction. Inadequate knowledge may lead to the patients’ misinterpreting their own symptoms, subsequently furthering delay in decision making for seeking proper treatment (24). Our study found that patients who perceived their symptoms as cardiac in origin had significantly shorter symptom-to-door times (*P* = 0.005). These findings agree with previous studies, which also found that cognitive factors, such as the patients’ own perceptions of their symptoms as being cardiac related, affect their decisions to seek treatment earlier (9, 19, 24). Therefore, further investigations on decision making processes are needed to gain more insight into the reasoning behind this.

We also determined that patients with recurrent chest pain or similar presentations tend to delay more compared with patients for whom these symptoms are first presenting. This predictor was also found to be significant for pre-hospital delay among STEMI patients in previous studies (19, 22). This paradoxical finding may be explained, firstly, by patients tending to believe that their symptoms are transient and will go away after resting and self-medication, such as sublingual nitrates and oral analgesics (26). Khraim and Carey (22) concluded that patients’ beliefs may also be linked with some psychological factors, such as denial of myocardial infarction, fear of the consequences and fear of troubling others—especially in those patients with poor social support. Secondly, previous chest pain episodes may have been intermittent, dull in nature and less severe—improving after rest or oral medications. In 2013, McKee et al. found that symptoms that were continuous and sudden-onset were associated with shorter delay times compared with intermittent and gradual-onset symptoms (9). In our univariate analysis, severe chest pain, with a pain score of seven to ten, had lower odds of delaying emergency department arrival (*P* = 0.003), while the effects of mildly and moderately severe chest pains were not significantly different from patients with no chest pain in terms of symptom-to-door time. This finding signified that patients tend to delay in making the decision to seek treatment if the chest pain is not severe enough.

### Places of Symptom Onset

With respect to places of symptom onset, 73.4% of our studied population first experienced symptoms at home; 12.6% were at their workplaces; and 14% were in public places. We found that patients who experienced chest pain in public places had significantly lower odds of delaying emergency department arrival compared with people who experienced chest pain at home (*P* = 0.010). Our findings were similar to some previous studies, in which the authors also determined that, if symptoms begin when patients are at home and alone, patients tend to delay more, compared to symptoms beginning when patients are in public places with bystanders (27–29). Indeed, a more recent study by Coventry et al. (11) noted that decisions to call for an ambulance from home, by anyone other than the patient’s spouse, were associated with increased pre-hospital delay. Occurrence of symptoms in public places likely alarms bystanders, spurring them to assist patients in seeking earlier treatment by sending patients to the nearest hospital or making an ambulance call. However, when at home, patients tend to self-medicate and rest, hoping the symptoms will go away, thus delaying symptom-to-door time.

### Means of Transportation

We also examined the relationship between pre-hospital delay and means of transportation. Among our study population, 147 (66.2%) were sent to the emergency department by another person; only 66 (29.7%) patients were transported by ambulance; and nine (4.1%) patients drove themselves to the hospital. This finding signifies underutilisation of ambulance services among STEMI patients in Malaysia compared to other Western countries, which documented 62.5% to 63.1% of myocardial infarction patients being brought to hospitals via ambulance (11, 30). This is probably due to misconceptions in Malaysia regarding long wait times for ambulances, leading people to presume that patients will reach a hospital faster via their own modes of transportation, without considering the resuscitation facilities available in ambulances if patients are hemodynamically unstable. Nonetheless—as opposed to many previous studies (9, 11, 19, 29, 30), which found that ambulance usage significantly shortens pre-hospital time—our study found that the means of transportation were not statistically significant in predicting pre-hospital delay. Among our studied population, patients who were sent to a hospital by ambulance were mainly sent by health clinics (48.5%) and GP clinics (22.7%). Only 19 (28.8%) out of 66 ambulance users actually made an ambulance call directly. This FMC connection may explain the prolonged symptom-to-door time, despite patients being sent to the hospital via ambulances.

## Limitation

Patients may have recall bias regarding the exact time of symptom onset. This is especially true of data collected from family members when patients were too critically ill, or had passed away, and could not provide information themselves. Some eligible patients were also discharged prior to the seven-day data collection period ending, and, therefore, there was discontinuity in the data collection. Additionally, samples were only gathered from Kubang Kerian and Kuala Terengganu, which might cause sample bias and affect the generalisability of these results to other settings in Malaysia.

## Conclusion

Our study revealed the following four factors associated with prolonged symptom-to-door time as predictors of pre-hospital delay: female gender, recurrence of chest pain, atypical symptoms and choosing to see a GP as FMC. Nonetheless, three factors—hyperlipidaemia, symptoms occurring in public places and patients self-perceiving their symptoms as cardiac related—shortened symptom-to-door time among STEMI patients. Surprisingly, ambulance usage was not a factor affecting symptom-to-door time in our study. These findings illustrate the need for future education to raise awareness of pre-hospital delay among higher risk groups of the Malaysian population. In addition, ambulance services are insufficiently used by Malaysian patients experiencing chest pain, and this may require further, detailed studies to reveal modifiable factors associated with this underutilisation. Future qualitative study to explore levels of myocardial infarction knowledge, related cognitive factors and decision-making processes in the Malaysian population may also be necessary for determining other factors associated with symptom-to-door time.

## Figures and Tables

**Table 1:** Comparison of baseline characteristics for STEMI patients presented early and late (*n* = 222)

Variables	≤ 3 h *n* (%)	> 3 h *n* (%)
**Sociodemography**		
Age (years)^a^	56.87 (11.53)	59.98 (12.33)
Gender		
Male	129 (90.8)	57 (71.3)
Female	13 (9.2)	23 (28.7)
Education level		
Primary school	44 (31.0)	31 (38.8)
Secondary school	74 (52.1)	39 (48.7)
Tertiary school	24 (16.9)	10 (12.5)
**Risk factors and comorbidities**		
Hypertension		
Yes	65 (45.8)	53 (66.2)
No	77 (54.2)	27 (33.8)
Diabetes mellitus		
Yes	47 (33.1)	35 (43.8)
No	95 (66.9)	45 (56.2)
Family history		
Yes	42 (29.6)	15 (18.8)
No	100 (70.4)	65 (81.2)
Hyperlipidaemia		
Yes	54 (38.0)	23 (28.8)
No	88 (62.0)	57 (71.2)
Smoking		
Yes	94 (66.2)	40 (50.0)
No	48 (33.8)	40 (50.0)
**Clinical characteristic**		
Previous episode of chest pain		
Yes	60 (42.3)	43 (53.8)
No	82 (57.7)	37 (46.2)
Chest pain		
Typical	127 (89.4)	55 (68.8)
Atypical	15 (10.6)	25 (31.2)
If chest pain present, severity (pain score)		
No pain [0]	9 (6.3)	12 (15.0)
Mild [1–3]	6 (4.2)	6 (7.5)
Moderate [4–6]	24 (17.0)	29 (36.3)
Severe [7–10]	103 (72.5)	33 (41.2)
Self-perceived cardiac symptoms		
Yes	83 (58.5)	22 (27.5)
No	59 (41.5)	58 (72.5)
Places of symptom onset		
Home	94 (66.2)	69 (86.2)
Workplace	21 (14.8)	7 (8.8)
Public place	27 (19.0)	4 (5.0)
**First medical contact (FMC)**		
Emergency department	108 (76.1)	44 (55.0)
Health clinic	20 (14.1)	14 (17.5)
General practitioner	14 (9.8)	22 (27.5)
**Means of transportation**		
Ambulance	43 (30.3)	23 (28.8)
Sent by another person	95 (66.9)	52 (65.0)
Self-drive	4 (2.8)	5 (6.2)

a Mean (SD = Standard deviation)

**Table 2:** Factors associated with pre-hospital delay among STEMI patients (*n* = 222) (simple logistic regression)

Variables	Crude B	Crude OR (95% CI)	Wald	*P*-value

Sociodemography				
Age	0.02	1.02 (0.99, 1.05)	3.44	0.064
Gender				
Male		1		
Female	1.39	4.00 (1.90, 8.46)	13.2	< 0.001
Education level				
Primary school		1		
Secondary school	−0.29	0.75 (0.41, 1.37)	0.89	0.344
Tertiary school	−0.53	0.59 (0.25, 1.41)	1.40	0.236
**Risk factors and comorbidities**				
Hypertension				
Yes	0.84	2.33 (1.32, 4.11)	8.45	0.004
No		1		
Diabetes mellitus				
Yes	0.452	1.57 (0.90, 2.76)	2.48	0.115
No		1		
Family history				
Yes	−0.60	0.55 (0.28, 1.07)	3.10	0.079
No		1		
Hyperlipidaemia				
Yes	−0.42	0.66 (0.36, 1.19)	1.93	0.164
No		1		
Smoking				
Yes	−0.67	0.51 (0.29, 0.89)	5.54	0.019
No		1		
**Clinical characteristic**				
Previous episode of chest pain				
Yes	0.46	1.59 (0.92, 2.76)	2.71	0.100
No		1		
Chest pain				
Typical		1		
Atypical	1.35	3.84 (1.88, 7.86)	13.68	< 0.001
If chest pain present, severity (pain score)				
No pain [0]		1		
Mild [1–3]	−0.29	0.75 (0.18, 3.12)	0.16	0.692
Moderate [4–6]	−0.10	0.91 (0.33, 2.51)	0.04	0.850
Severe [7–10]	−1.43	0.24 (0.09, 0.62)	8.67	0.003
Self-perceived cardiac symptoms				
Yes	−1.31	0.27 (0.15, 0.49)	18.74	< 0.001
No		1		
**Places of symptom onset**				
Home		1		
Workplace	−0.79	0.45 (0.18, 1.13)	2.89	0.089
Public place	−1.60	0.20 (0.07, 0.60)	8.20	0.004
**First medical contact (FMC)**				
Emergency department		1		
Health clinic	0.54	1.72 (0.80, 3.70)	1.91	0.167
General practitioner	1.35	3.86 (1.81, 8.22)	12.24	< 0.001
**Means of transportation**				
Ambulance		1		
Sent by another person	0.004	1.00 (0.55, 1.85)	0.00	0.991
Self-drive	0.849	2.34 (0.57, 9.56)	1.39	0.238

OR = Odds ratio, CI = Confidence interval

**Table 3:** Factors associated with pre-hospital delay among STEMI patients (*n* = 222) (multiple logistic regression)

Variables	Adj B	Adj OR	95% CI	Wald	*P*-value
Gender					
Male		1			
Female	0.88	2.42	(1.02, 5.76)	3.98	0.046
Hyperlipidemia					
Yes	−0.78	0.46	(0.23, 0.93)	4.70	0.030
No		1			
Previous episode of chest pain					
Yes	1.01	2.74	(1.37, 5.46)	8.20	0.004
No		1			
Chest pain					
Typical		1			
Atypical	0.97	2.64	(1.11, 6.31)	4.80	0.029
Self-perceived cardiac symptoms					
Yes		1			
No	−1.04	0.36	(0.17, 0.73)	7.81	0.005
Places of symptoms onset					
Home		1			
Work place	−0.53	0.59	(0.21, 1.65)	1.02	0.313
Public place	−1.57	0.21	(0.06, 0.69)	6.64	0.010
First medical contact (FMC)					
Emergency department		1			
Health clinic	0.13	1.14	(0.46, 2.79)	0.08	0.782
General practitioner	1.03	2.80	(1.20, 6.56)	5.64	0.018

Notes: Multicollinearity and interaction terms were checked and not found. Hosmer-Lemeshow test (*P* = 0.729). Classification table is 64% and predicted probability with ROC curve 80.1% (95% CI: 74.3%, 86%, *P*-value < 0.001).
